# Ethics and regulation of neuronal optogenetics in the European Union

**DOI:** 10.1007/s00424-023-02888-8

**Published:** 2023-11-24

**Authors:** Timo Faltus, Johannes Freise, Carsten Fluck, Hans Zillmann

**Affiliations:** Law School, Faculty of Law, Economics and Business, Martin Luther University Halle-Wittenberg, Halle, Germany

**Keywords:** Neuronal optogenetic, Cerebral organoids, Ethics, Law, Regulation, Translation

## Abstract

Neuronal optogenetics is a technique to control the activity of neurons with light. This is achieved by artificial expression of light-sensitive ion channels in the target cells. By optogenetic methods, cells that are naturally light-insensitive can be made photosensitive and addressable by illumination and precisely controllable in time and space. So far, optogenetics has primarily been a basic research tool to better understand the brain. However, initial studies are already investigating the possibility of using optogenetics in humans for future therapeutic approaches for neuronal based diseases such as Parkinson’s disease, epilepsy, or to promote stroke recovery. In addition, optogenetic methods have already been successfully applied to a human in an experimental setting. Neuronal optogenetics also raises ethical and legal issues, e.g., in relation to, animal experiments, and its application in humans. Additional ethical and legal questions may arise when optogenetic methods are investigated on cerebral organoids. Thus, for the successful translation of optogenetics from basic research to medical practice, the ethical and legal questions of this technology must also be answered, because open ethical and legal questions can hamper the translation. The paper provides an overview of the ethical and legal issues raised by neuronal optogenetics. In addition, considering the technical prerequisites for translation, the paper shows consistent approaches to address these open questions. The paper also aims to support the interdisciplinary dialogue between scientists and physicians on the one hand, and ethicists and lawyers on the other, to enable an interdisciplinary coordinated realization of neuronal optogenetics.

## Introduction

Neuronal optogenetics raises (medico-) ethical and legal questions as well as questions of social acceptance due to the investigated objects, the methods of research, and the possible application in humans. The biotechnological approach of optogenetics is to specifically control cellular activities by light. For this purpose, it is necessary to first (trans-/xeno-) genetically modify the neuronal cells to be influenced later so that they express light-sensitive ion channels or other transporters, particularly in the cell membranes [12]. The key breakthrough came with the discovery that light-gated ion channels (so-called channelrhodopsins) from algae can be integrated into cells of other organisms, including mammals and human cells, making them light-sensitive [44]. The controllability of cells genetically modified in this way is based primarily on the fact that the naturally occurring and variable electrical polarity of the cell can be specifically influenced by the light stimulation of the optogenetic process. With the use of natural channelrhodopsins and the development of genetically modified channelrhodopsins, optogenetics has advanced significantly.

By combining the methods of genetics and optics, optogenetics can be used to study the cell physiology in a cell-type specific manner, e.g., in neuronal cells, especially neurons in cell cultures as well as in animal models [30]. In basic research, neuronal optogenetics approaches are expected to advance knowledge about the functions and controllability of specific neuronal classes. Optogenetic methods can also be applied to stem cells [45] or muscle cells [68]. However, the application of optogenetics to neuronal cells (neuronal optogenetics) is of greater practical interest than the application to other cells, as neuronal optogenetics is intended to be applied to humans as a therapy for neuronal diseases [30]. In the last decade, the development of optogenetics has made progress using it in human cerebral organoids and in animal models, so that a transfer to human medicine is being discussed [30]. In the meantime, optogenetic methods have been used to partially recover visual function in a blind patient [52].

However, given the technical successes of optogenetics, why do ethical and legal questions arise about this technology? Specifically, research and application of optogenetics in animal models raise questions of animal ethics as well as questions about the regulatory framework of animal experiments for research on optogenetic issues. The further development of cerebral (human) organoids may open up a new sub-discipline of neural optogenetics. This could expand the current research radius of neuronal optogenetics, which has so far been based mainly on the viral introduction of optogenetic DNA vectors into animals or the use of transgenic animals. However, the generation and use of human cerebral organoids has raised questions about the ethical and legal status of these organoids, among other questions [24, 31, 64, 66, 74, 76]. The answer to this question has an influence on the question in which way cerebral organoids can or may be handled. Are cerebral organoids ethically and legally ordinary things, such as any laboratory equipment, or do cerebral organoids have a different ethical or legal status, which might allow only a restricted use compared to things.

Ethical and legal questions also arise regarding the (future) application of optogenetics in humans. The medico-ethical issues concern questions of patient autonomy and of the balance of safety and benefit of the application for patients, as well as questions of access and justice. From a legal perspective, it has not yet been clarified whether the methods of neuronal optogenetics are adequately covered by pharmaceutical law and medical device law to successfully translate this technology into medical practice. Regarding the economic protection of the therapeutic application of optogenetics, questions also arise as to the extent to which inventions for the therapeutic use of optogenetics can be protected by patent law.

In view of the technical possibilities of optogenetics and especially in view of the use of cerebral organoids, scientists as well as legal scholars and ethicists have pointed out these open ethical and legal questions and suggested their clarification [e.g., 10, 13, 18, 29]. The article describes the open ethical and legal questions of optogenetics and the extent to which these open questions affect the translation of optogenetics from basic research into medical practice. The article also shows first, not yet final, approaches to answering these open questions. The paper particularly addresses the scientific, medical and technical communities and aims to raise their awareness for the ethical and legal issues of this technology, to promote the interdisciplinary discourse that is necessary for a smooth translation of optogenetics.

## The interplay of ethics and law in the appraisal of new technologies

Optogenetics and the translation of optogenetics can benefit from accompanying ethical and legal research, since the translation of a technology requires an ethical backup as well as the outline of a legal framework specific to the technology. Weighing the consequences of technical innovation therefore increasingly requires that the impacts of this technology be considered holistically and not limited to the merely technical benefits. In addition to technical research, especially ethical and legal accompanying research is carried out. Accompanying research therefore provides an essential contribution to the translation of a new technology.

Regarding accompanying research on ethical and legal issues, it should be noted that the research of these two disciplines in turn benefits from each other in terms of consistent accompanying research. This is because although ethics and law are different disciplines, they are nevertheless interconnected.

Ethics and law—and thereby the corresponding accompanying research—are interconnected because ethics provides the moral foundations for the laws of a society. Legal systems are based on moral principles and values that are considered important in society. An interdisciplinary collaboration between ethics and law can help ensure that laws reflect ethical standards and moral principles. In addition, ethical issues arise in numerous areas of law. Ethicists and lawyers can work collaboratively to examine these issues and find consistent answers. Further, ethics can define general principles and values, while law establishes specific rules and regulations. Interdisciplinary collaboration between ethics and law can also help clarify how ethical principles can be translated into specific legal provisions. Finally, a coordinated dialogue between ethics and law can help identify and resolve potential conflicts between legal requirements and moral convictions. Overall, cross-disciplinary collaboration between ethics and law facilitates a more comprehensive and balanced consideration of legal issues and helps to promote fairer and more ethical legislation and legal practices. This mutual benefit of both disciplines can therefore also be achieved in the interdisciplinary study of neuronal optogenetics.

## Ethics of optogenetics

### Protective status of cerebral organoids in research

Within the ethical debate on the evaluation of cerebral organoids, it has been argued that against the background of further technical developments it might be necessary to grant an ethical protective status to cerebral organoids, as they might for example have pain perception in the future [33]. This is because further technical development could also lead to a greater practical significance of cerebral organoids in research and application. From an ethical perspective, calls for a protected status are made when the challenged entities have certain capacities such as pain perception or even forms of consciousness [33]. Such protected status could impact the ability to use cerebral organoids in research and application if, for example, ethics committees oppose research or application of the technology. In addition, societal acceptance could also decline due to emerging ethical issues. Both negative votes from ethics committees and insufficient societal acceptance could hinder scientific research. It is therefore necessary to develop appropriate recommendations for action timely. This is because the morally desirable potential of technology in relation to the therapy of sick people not only obliges us to take a critical approach to technology and possible aspects of uncertainty in its application. From an ethical perspective, they also oblige us to investigate under which circumstances the application of the technology to humans can be approved on ethical grounds. Cerebral organoids have already been used to investigate how the Zika syndrome affects the fetal development of the brain, or how microcephaly can be better understood [14]. It is also possible to test both the toxicity and effectiveness of drugs [14]. A future goal is to obtain parts of brain tissue that can by transplantation replace damaged parts of the brain in patients [6, 60]. Against the background of these possibilities, there is therefore a moral obligation to continue this research and thus make its potential usable, as well as an obligation to ethically safeguard the research and application of optogenetics. Ethical safeguarding is essential if the technology is to be transferred to clinical application.

Despite the limitations in size and lifespan of the organoids against the background of a lack of vascularization, properties of the human central nervous system could already be realized in cerebral organoids: Thus, cerebral organoids have autonomous electrical activity, and one study was able to show that EEG patterns comparable to those of premature human infants are evident [67]. It can be concluded that even in the absence of external stimuli, neuronal activities comparable to those of a human brain are generated [33]. Here, too, it must be ethically examined which conclusions can be drawn regarding the ethical status of human cerebral organoids. The question arises as to what further developments research on and with cerebral organoids will yield and what properties or capabilities cerebral organoids will develop in the further course. From an ethical point of view, the lack of knowledge about the further development of cerebral organoids can be criticized, as well as the difficulty to reliably measure or recognize the consciousness or other functions or abilities developed in a cerebral organoid. For even if it were assumed that cerebral organoids can form basal forms of consciousness, the question would remain to be answered as to how their presence can be detected.

### Epistemic objections

Objections to emerging technologies that concern knowledge or non-knowledge are nothing new. Already from the debates about green and red genome editing, arguments are known that refer to knowledge or non-knowledge about the short-, medium-, and long-term consequences of an application of the technology in question [81]. Green genome editing refers to the use of genetic engineering techniques in agriculture, particularly in plants, while red genome editing is defined as the use of these techniques in human medicine. In connection with this, there is always the question of the degree of knowledge about the relationship between the risks and benefits of a technology that justifies its use. It can be stated that conclusive knowledge about any area of the world will probably never exist. Nor about the possibility of cerebral organoids developing abilities that would make us think about granting them a moral status based on their capacity for suffering or even consciousness. For it must be stated at this point that it makes a difference whether a living being can percept pain or realizes higher functions of consciousness. An argument for the fact that research on and with cerebral organoids is ethically not acceptable cannot be based on insufficient knowledge. For in this context, the question also arises as to whether a renunciation of the possible potentials can be ethically justified by incomplete knowledge. In this context, only precise technology assessments can be called for. Insofar as the clinical or therapeutic application of organoid technology pursues the goal of reducing or preventing human suffering and thereby increasing human life chances, attention must be paid above all to compliance with medico-ethical standards.

### Consciousness, human consciousness, and organoids with consciousness

The argument about the moral status of cerebral organoids also concerns questions about their level of organization, because it is not up for discussion that human consciousness is the highest form of an ability that other living beings on this planet also possess. The emergence of consciousness is explained by an evolutionary selection principle [10, 16]. Against this background, the question arises whether, at this point in time, all living beings that have a basal consciousness are actually part of human moral considerations. One could easily undertake reflections on the scientific instrumentalization of animals here. Now, it is not fair to play off the protective status of one group of entities against that of the other. For this reason, it will not be shown here how contradictory it appears when, on the one hand, it is considered to what extent and, above all, from what stage of development human cerebral organoids require a protective status when other animate entities, about whose capacity for perception of pain there is no doubt, are used in scientific research. Should the central problem that is seen in the debate be the fact that human cerebral organoids are being discussed? Proposals to give cerebral organoids the legal status of animals in research are on the table [74]. The ethical status of human cerebral organoids remains unresolved. The need for an interdisciplinary debate in a timely manner is seen [54, 57, 74]. And indeed, ethical and legal reflection would do well to accompany the development of techniques such as optogenetics or organoid technology as far as possible from the outset. Not only can risks possibly be identified in this way that cannot always be seen in the scientific debates. The translation of basic research into applied human medicine can also be facilitated by accompanying ethical research.

### Neurophysiological level of organization and further conditions for consciousness

There is no doubt that the human brain is a necessary condition for all mental states in which people can be in. The experience of pleasure and suffering, the experience of an own and private ego instance and connected with it the remembering, feeling, but also the most complex thinking about difficult facts would not be possible for humans if the human brain had not reached the organizational level it has during an evolutionary process.

However, at least two further factors appear to be of importance for human consciousness: (1) The human brain is located in a body that enables the brain to interact with its environment and the objects in it [9]. This exchange shapes the brain, as the complex interplay between the brain and its surroundings influences cognitive development and sensory perceptions. [50]. (2) Humans live in social associations that enable development, which is called culture. Human culture and the human brain are interrelated because human culture shapes the environment in which humans or their brains develop. And the objects of employment are also created to a considerable extent by human cultural activity. Both aspects can be seen as the condition of possibility for the kind of consciousness that is present in humans. Cerebral organoids will be not able to fulfill both conditions in the foreseeable future.

A sensorium, which enables contact with the environment, is by no means sufficient. For the perception of pain, for example, certain neurophysiological conditions must be present in the corresponding brain and in the body to enable the underlying stimulus to be transmitted or processed. An evaluation of the stimulus then only turns it into pain in the actual sense, which, for example, a person feels or perceives qualitatively. It is therefore not excluded that cerebral organoids could process and evaluate stimuli, which would then be approximately what humans experience as impressions between pleasure and displeasure. With regard to cerebral organoids, the question arises what status they would have ethically and legally if they could percept something like pain.

### The moral status of genetic engineering interventions between naturalness and artificiality

At the level of principles, there are arguments that are based more on the cosmological and ideological attitudes of their authors than on ethically founded insights. For example, it could go against various values to intentionally perform genetic engineering interventions on humans. *Values of life*, of *divine creation*, or of *naturalness* could be mentioned here [81]. This last value will be discussed here: Naturalness as a normative value cannot be considered without its conceptual counterpart, artificiality. Particularly prominent within the cultural philosophical tradition is a distinction between the two concepts on the following basis:

All entities are regarded as natural which have originated or originate without human intervention, as artificial again those entities are considered which were created intentionally or were brought into the world. Generally, one assumes in this connection, even if not undisputed, that only humans create entities intentionally, i.e., that only humans create artificial. It can now be stated that people encounter naturalness with a moral bonus, which leads them to the conviction that natural entities are less dangerous than artifacts [4]. The latter are met with skepticism regarding safety and the possible dangers they could pose. Similarly justified concerns have already been raised about green and red genetic engineering [81]. Now there is no need for philosophical reflections on the fact that the dangerousness of a poison depends on its effect, e.g., on humans, and not on the origin of the poison from nature or a laboratory. There is no doubt that a multitude of ethically desirable human interventions in nature can be enumerated: Medical measures, nature reserves, etc. could be cited here. More profound statements about the question, why the natural for the human being is estimated in such a way, are not to be made here.

At this point, it should be added that it remains to be investigated whether organoids possibly defy categorization as natural or artificial. This is because the cell assemblies are created by autonomous division, while at the same time the actual process is initiated by humans. However, since, as explained, categorization has no ethical significance, it does not seem necessary from an ethical point of view to determine the extent to which organoids represent natural or artificial entities.

### The perspective of animal ethics

One application of neuronal optogenetics has animal ethical implications: Animal experimentation is related to questions about the moral status of animals, the human-animal relationship, and the treatment of animals by humans [51]. Animal experiments are a necessity if basic research on neuronal optogenetics is to be transferred to human application. Therefore, in the context of neuronal optogenetics, the question is not whether animal experiments are necessary, but only how they should be ethically evaluated and how or whether they can be ethically justified. There is a need for ethical research because neuronal optogenetics aims to manipulate animals not only physically but also in their psyche. Furthermore, the debate has changed in recent years and the rights of nature and animals are being re-evaluated in light of biocentric arguments. This change concerns the transition from anthropocentric morality to the notion that animals have their own moral rights. The questions that arise in this context are as follows: (1) how can the interests of humans and animals be mutually balanced in the context of neuronal optogenetics? Within the discourse, there is a debate between anthropocentrism, physiocentrism, and preference utilitarianism. While anthropocentrism claims that only humans are morally relevant, physiocentrism goes beyond this and claims the intrinsic moral value of all components or the totality of nature. Preference utilitarianism, in turn, assumes that only living beings with preferences have intrinsic moral value. Against this background, the preferences of animals and humans would have to be weighed against each other. In this respect, the existing arguments have to be weighed and, if necessary, new arguments must be developed with respect to neuronal optogenetics. (2) What knowledge gain and transferability can be expected from animal experiments in neuronal optogenetics and how should they be morally evaluated? This question is relevant to justify the necessity of animal experiments. (3) What ethical recommendations can be developed for the use of animals for neuronal optogenetics in basic and preclinical research? Again, further ethical research is needed. (4) To what extent can animal replacement by organoids be achieved in basic research? This would represent a desirable outcome from an animal ethics perspective, as the instrumentalization of animals in research holds potential for ethical conflict.

### Medico-ethical considerations

Prognostically, there is also a need for ethical research regarding an application of neuronal optogenetics in human medicine. Only in this way can a translation of research from basic research into clinical application be ethically secured and thus made possible. Among other things, it seems appropriate to conduct this against the background of the four medico-ethical standards (autonomy, non-maleficence, beneficence, and justice). This approach gives rise to the following complex of questions:

1. What effects does the application of organoid technology have on human autonomy or patient autonomy? In this context, it is necessary to investigate the impact of therapeutic applications of neuronal optogenetics in the human brain on personality, freedom of choice and autonomy. It should be noted that therapeutic approaches aim to change the mental state of patients, e.g., in the therapy of depression and post-traumatic disorders. Therefore, on the one hand, it is necessary to ethically examine and evaluate the possible effects on the autonomy or freedom of patients. Is it even possible to consent to an intervention that has the goal of changing one’s personality? On the other hand, this raises fundamental philosophical questions about the relationship between brain and personality, consciousness, authorship, and self-perception and perception by others. It is to be expected that such interventions will not be understood as an attack on the patient’s autonomy, but as an attempt to restore the patient’s autonomy through therapeutic intervention. However, this makes it necessary to clearly define what can and cannot be counted as a therapeutic intervention.
  In addition, it should be noted that an initial application of neuronal optogenetics could be the treatment of neurodegenerative diseases such as Tourette’s and Parkinson’s diseases [47]. Preserving the principle of autonomy in individuals with neurodegenerative diseases may be associated with additional difficulties that require ethical consideration [27]. The positive interpretation of the autonomy principle places the decision-making autonomy of the individual in the foreground. From this, a plea can be derived for respect for autonomous decisions for new and high-risk treatments, even if these are viewed skeptically by medical experts under certain circumstances [70].
2. Do both research and application of neuronal optogenetics live up to the principle of non-maleficence? Here, above all, it must be examined in a forward-looking manner whether the actions of the actors follow the principle of *primum non nocere*. This reflection is future-oriented, since above all the perspectives of a medical application of the technology must be examined. Against this background, it must be investigated how, with regard to the immediate goals, such as use in observing and studying the work of neurons or neuronal associations, and also the distant goals, such as application to alter the physiology of neuronal cells in humans, it can be ensured that both individual normative judgments can be taken into account and (utilitarian) cost–benefit balances can be drawn up. A current cost–benefit balance is thereby characterized by an uncertain data situation of the risks of viral gene transfer in the brain, which cannot be outweighed by the uncertain prospect of efficacy [70].
3. Are the actions of researchers and physicians fundamentally compatible with the principle of beneficence? Medico-ethical reflection must raise the question of the extent to which the principles of beneficence, life, health, and quality of life are guaranteed by actions of researchers and physicians. Are the goals of research or application compatible with the human preference for avoiding suffering? The principle of care must be examined here for the possibility of coming into conflict with the principle of (patient) autonomy, since the two principles might suggest different actions in certain cases of medical application. In such cases, it would be necessary to weigh both principles against each other and to hierarchize them.
4. What is the relationship between the clinical application of neuronal optogenetics and the principle of justice? The question here is, among other things, whether an application of the neuronal optogenetic technique can be expected to result in a fair and appropriate distribution of the health benefits arising from this application. The admissibility of costly therapies can also be examined in this context. It is likely that securing an ethical viability in terms of equity standards will be a matter of legislative regulation. However, the need for such legislative regulation can be worked out through ethical analysis.
5. What dangers of dual use are to be expected from neuronal optogenetics and how are they to be dealt with ethically? The ethical analysis must also address the question of possible clinical and non-clinical applications of neuronal optogenetics that do not pursue the goal of concrete therapies, but rather use the technology in the military field, for example. These ethical questions must be brought closer to an answer if the morally desirable potentials of the technology are to benefit patients in the form of therapeutic approaches.

## Regulation of optogenetics

### Systematic legal analysis for translation

From a legal point of view, the successful transfer of basic research results into regular medical application (so-called translation) requires that the legal questions of a (new) technology and thus also of (neuronal) optogenetics have been systematically identified from basic research to application and that it has been clarified how these questions can be answered consistently. This is particularly due to the fact that a legal obstacle to the translation in terms of a legally unclear application at the end of the translational process, i.e., with regard to its medical application, can lead to basic research and applied research not taking place and to investments in this research not being made because the legal possibility of the translation is not guaranteed. Vice versa, legally unclear questions of basic research and applied research may lead to a situation where this research, despite its therapeutic potential, does not take place or possibly shifts to legal systems where these questions have already been answered. Since a consistent legal analysis for the entire translational process of neuronal optogenetics is not yet available, from a legal point of view, neuronal optogenetics faces the risk that translation will not be successful due to unanswered legal questions.

The methods of neuronal optogenetics are used today in basic research as well as in preclinical studies. In this context, these methods are used in 3D cell models (in vitro) [20, 34, 42, 58], especially cerebral organoids [42, 58], or animal models [43, 71]. In both fields, as in the context of ethical analysis, independent but nevertheless interrelated legal questions arise, for example concerning the (legal) status of cerebral organoids or the handling of laboratory animals. Given the results already achieved, especially in studies on neuronal optogenetics in animals, the medical application of neuronal optogenetics in humans is already being discussed [1, 72, 73]. The partial recovery of visual function using optogenetic methods in a blind patient is a first clinical success of optogenetics [52]. However, due to the higher complexity of the combination of genetic intervention and neuronal stimulation compared to established methods of gene therapy or deep brain stimulation, the considerations on the legal classification of deep brain stimulation [28, 39, 49, 55] cannot directly be applied to neuronal optogenetics.

In the following, the legal questions concerning the translation of neuronal optogenetics as well as the relevant legal regulations are introduced. In addition, initial approaches to addressing these hitherto unanswered questions will be outlined.

### Relevant fields of law and legal sources

The translation of neuronal optogenetics from basic research into medical practice is governed by several areas of law, such as genetic engineering law, pharmaceutical law, and medical device law, as well as patent law. On the one hand, the law of the European Union (EU) and other international legal documents play a role within these areas of law, on the other hand, the law of the respective EU Member State. In the following legal description, the legal framework of neuronal optogenetics is explained based on the requirements of EU law, which thus applies in all EU Member States. However, a distinction must be made between EU regulations and EU directives. Regulations from EU directives must be adopted by the member states in national law. In contrast, requirements from EU regulations have direct legal effect for public and private actors throughout the EU. In addition, the significance of other international legal documents relevant to optogenetics is presented in the following legal assessment of optogenetics. Exemplary national legal requirements are explained using the case of Germany.

### Optogenetic research on cerebral organoids

The advancement of cerebral (human) organoids can open up a new branch of research for neuronal optogenetics, which has its own legal questions. Until today, neither EU law nor other international legal documents [69] impose any explicit restrictions on research on human cerebral organoids. Therefore, the use of optogenetic methods on cerebral organoids is not subject to any specific regulation under European law or international law. Currently, there are also no efforts known to establish such a legal framework to deal with cerebral organoids. Calls for an international (soft law) declaration to regulate the use of cerebral organoids [69] have not yet been implemented. The same applies to national legislation. Specific sub-constitutional law regarding (cerebral) organoids does not exist in German law [64–66] and, to the best of our knowledge, do not exist in any other European state. However, the lack of regulations with explicit reference to (cerebral) organoids does not mean that (cerebral) organoids are in a legal vacuum.

The question of the legal framework for the generation and use of cerebral organoids is significantly determined by the legal status of these entities. In addition to the discussion on the moral status of cerebral organoids presented in the ethical part of this paper, a parallel discussion is also ongoing in the field of law. The arguments in the ethical and legal discourse revolve equally around potential consciousness and sensitivity to pain of the organoids [31, 76]. However, at this point, it must be emphasized that ethical concerns do not necessarily lead to a legal restriction or their predominance over protected legal positions.

The question of the legal status of cerebral organoids concerns the clarification of whether cerebral organoids are things, i.e., res (legal objects) under current law or whether cerebral organoids already have a legal status that goes beyond, whereby the generation and use of cerebral organoids would possibly be legally restricted compared to things. In terms of an evidence-based technology assessment, this legal assessment also must consider technical progress, e.g., the fact that future cerebral organoids may have physiological capabilities that are not present in today’s cerebral organoids.

No specific (protective) status of cerebral organoids results from the EU Charter of Fundamental Rights (EU CFR). Due to the different legal traditions of the EU member states with respect to the protection of dignity, only the protection of born human beings can be indisputably derived from the human dignity guarantee of Article 1 EU CFR (the discussion about this is still ongoing) [26]. Consequently, other entities, such as human cerebral organoids, are not covered by the scope of protection of Article 1 EU CFR. Furthermore, organoids are also not body parts protected under Article 3(2) EU CFR [64–66]. Due to the lack of EU law and the absence of further requirements under international law, the question of whether they are to be treated as things must therefore be derived from national law.

In countries with a constitution that provides a specific protection of human dignity, such as Germany, the question of whether legal restrictions on the handling of cerebral organoids of human origin can be based on this is discussed but cannot be justified persuasively according to the current state of the development of organoids [19, 64–66]. Among other things, this can be explained by the fact that cerebral organoids do not have the potential to develop into a full human being [64–66, 76] or a holistic organism [19]. Despite the ethical arguments already mentioned, no other constitutional protection can be justified based on current law [74]. Cerebral organoids are in legal terms things i.e., res (legal objects) [19, 64–66]. The same applies to more complex assembloids that are composed of several cerebral organoids [19].

For the question of the legal handling of cerebral organoids, however, international and European sources of law on the one hand and national sources of law on biomedical and human genetic research on the other hand must be considered. Regarding the generation and use of cerebral organoids in basic research and preclinical studies, the general legal requirements resulting from regulations on the use of stem cells and genetic engineering must be observed in particular [19, 64–66, 76]. Depending on the possibilities of generating and using cerebral organoids, the requirements for informed consent (e.g., concerning its specificity) for cell donation in this regard must also be defined [13, 38, 62, 63, 74].

In view of the technical advances in the generation and research of cerebral organoids, however, future legislative reassessments of the generation and use of cerebral organoids are not generally ruled out. The regulations already discussed in the ethical and legal literature range from the obligation to have the research project evaluated in advance by an ethics committee [74, 76], to bans of certain methods. However, the scope of the constitutionally safeguarded scientific freedom must always be considered when legislating on potentially restrictive regulations for dealing with cerebral organoids [64–66]. This includes both, the European and the national levels. Freedom of research is enshrined in numerous international as well as national legal sources and is guaranteed in the EU according to Article 13 EU CFR.

### Optogenetics in animal models

For a comprehensive legal consideration, it is essential, among other things, to examine the legal relation between research on cerebral organoids and animal experiments [74]. The relevance of this is shown with respect to the internationally recognized 3Rs principle (replace, reduce, refine) laid down in Article 4 of Directive 2010/63/EU (directive on the protection of animals used for scientific purposes). As far as it is technically possible to replace animal experiments at least partially by research on (cerebral) organoids, it could therefore be legally mandatory, already according to today’s European legal situation, to avoid respective animal experiments [63, 74]. Additionally, legal aspects in the context of the transfer of human cerebral organoids to animals and the generation of chimeric organoids need to be examined [2, 19, 23, 74].

In the EU, legal regulations exist that base the legal classification as an animal experiment on the intervention in the animal (cf. Art. 3 No. 1 of Directive 2010/63/EU). In the case of an animal experiment within the meaning of the law, official permits may be necessary—depending on the type of animal and the context of the trial—to be able to legally conduct the experiment. Because of the partially invasive procedures of optogenetics, it can be assumed, even on a summary examination, that such studies on living animals are animal experiments as defined by the law. The implantation of cerebral organoids with optogenetic transformations in animals would represent a special case of optogenetic research from today’s perspective. However, in view of the legal basis outlined here, this would constitute animal experiments in the legal sense.

In the case of optogenetic research in the context of animal studies, the extent to which multilayered xeno-/transgenetic cholerization is legally justifiable must also be examined. This concerns, for example, the generation of xeno-/transgenic animals expressing a genetically modified light receptor of plant origin in neuronal cells or the transfer of human cerebral organoids—or parts of them—in which a genetically modified light receptor of plant origin, whose DNA has been incorporated into animal cells to generate the cerebral organoids, to an animal. The question of transferring genetically unmodified organoids to animals has already been discussed numerous times [19, 64–66, 74, 76]. Although there are various legal aspects to consider, there is no general prohibition [19, 23].

In view of this, it must be questioned whether the existing legal framework for regulating animal experiments, optogenetic studies, and studies on the implantation of cerebral organoids in animals adequately covers the opportunities and risks or whether there is a need for legislative action. It must therefore be clarified above all whether the permission, prohibition and official monitoring of such studies is appropriate regarding animal protection. Article 13 of the Treaty on the Functioning of the European Union (TFEU) establishes the consideration of animal welfare as a mandate for action for the EU and its Member States. However, because the scope of application of the provision is limited, it has only a minor legal effect [5]. In Germany, the constitutional enshrinement of animal protection in Article 20a of the Basic Law (Grundgesetz, i.e., the constitution) must be considered.

The recent approaches to the attribution of legally relevant dignity, corresponding dignity protection and rights of their own to non-human entities such as animals or nature as such, are discussed in the legal discourse as well as in the ethical discourse (see above) [3, 29, 61] but have not yet gained any practical legal significance.

### Application in humans

#### Medical device legislation

From a technical point of view, the therapeutic use of neuronal optogenetics is based on the stimulation of genetically modified cells with light. Accordingly, the development of suitable light sources, some of which are implantable, is a challenge in the context of neuronal optogenetics. The current work includes the further development of implantable micro-LEDs suitable for application to the human brain [22, 56]. To stimulate neuronal retinal cells, non-invasive devices such as special glasses [52] or headsets [59] may also be suitable.

Since the application of optogenetics on humans also requires the use of various objects and technical devices, a classification under medical product legislation is necessary. From a legal point of view, therapeutically used devices and objects may be medical devices. However, the use of medical devices requires that they fulfil the legal requirements for market access. Only such products may bear the CE (Conformité Européenne) mark, which indicates that the product in question fulfils the basic safety and performance requirements. Legal requirements for the manufacture, placing on the market and operation of medical devices, can be found in Regulation (EU) 2017/745, also known as the Medical Devices Regulation (MDR).

The MDR classifies medical devices into different risk classes. The classification is decisive for the requirements of the pre-market conformity assessment procedure. It is based on the health risks posed by a medical device when it is used for its intended medical purpose for patients. Among other things, the MDR differentiates between invasive and non-invasive as well as implantable and non-implantable devices.

Because optogenetic methods were not specifically considered in the legislative process for the MDR, it is necessary to review the extent to which the objects and technical devices used find an appropriate legal framework in the current MDR against the background of opportunities and risks or whether there is a need for regulatory reform.

According to the current legal situation, implantable micro-LEDs are active implantable medical devices in the sense of the MDR [72]. According to the status quo, these are regularly classified as medical devices of the highest risk class (class III), also due to their contact with the central nervous system. Within the framework of the conformity assessment procedure to be carried out according to the MDR the strictest requirements apply to them. In contrast, non-invasive devices for optogenetic stimulation are regularly assigned to class IIa or IIb. Compared to class III, they are subject to less stringent requirements for the review of the technical documentation [40]. In any case, however, the involvement of a supervisory authority (notified body) is mandatory for class IIa, IIb, and III devices. In contrast, a clinical investigation according to Article 61(4) MDR is in principle only mandatory for implantable devices and devices of class III.

#### Pharmaceutical law

According to the current state of technology, the therapeutic application of neural optogenetics is dependent on the genetic modification of the cells to be influenced by light. Genetic modifications in humans for therapeutic purposes are typically associated with questions of pharmaceutical law and genetic engineering law. Therefore, it must be clarified to what extent the genetic modification of human cells for medical purposes is regulated by pharmaceutical law and/or by genetic engineering law.

Under the condition that recombinant nucleic acid is required for the genetic transfer of the light-sensitive channelrhodopsins (virus vector including in particular suitable promoter sequence, sequences for ensuring transcription and translation, sequence for the required channelrhodopsins), this is to be classified under medicinal product law as a Gene Therapy Medicinal Product (GTMP). GTMPs fall within the scope of Regulation (EC) No. 1394/2007, also known as the Advanced Therapy Medicinal Products (ATMP) Regulation [72].

In principle, the manufacture of ATMP is only allowed in the EU if a manufacturing authorization has been granted by national authorities. The requirement and prerequisites of such a procedure result from Article 40–53 Directive 2001/83/EC. According to Article 46 Directive 2001/83/EC, the holder of the manufacturing authorization is obliged to comply with the principles and guidelines of the Good Manufacturing Practice (GMP). For the manufacture of ATMP, it is mandatory to follow the GMP guidelines specifically defined for ATMP by the EU Commission according to Article 6 Regulation (EC) 1394/2007.

Before being placed on the market, ATMPs generally also must undergo the central European authorization procedure according to Regulation (EC) No. 726/2004. The European Commission is responsible for the authorization decision, which is based on an authorization recommendation from the European Medicines Agency (EMA).

In contrast to genetic modifications in the context of animal studies, genetic modifications of humans are not covered by the genetic engineering legislation harmonized in the EU by Directive 2001/18/EC. Thus, medical applications on humans are also not regulated by genetic engineering law. According to the definition in Article 2(2) of Directive 2001/18/EC, a human being never becomes a genetically modified organism (GMO) in the sense of the law. A different question, however, is to what extent the application of a GTMP to humans requires an environmental risk assessment according to Directive 2001/83/EC in conjunction with Directive 2001/18/EC.

Regardless of the legal classification of neuronal optogenetics according to current law, however, there is also a need for further discourse as to whether the existing pharmaceutical law adequately cover optogenetics in view of the opportunities and risks or whether there is a need for regulatory change. In this context, also the possibilities of dual use and misuse, which have already been raised from an ethical point of view, must be covered by the future discourse [25, 41, 46, 78].

#### Informed consent

In the case of optogenetic interventions on humans, legal issues of informed consent are also relevant [72], because the comprehensive information of a patient or test person should enable a self-determined decision for or against an intervention. The requirement of the informed consent is generally recognized and safeguarded both in Article 3(2) EU CFR and in international agreements such as the European Bioethics Convention (Article 5(2)) and the UNESCO Declaration on Bioethics and Human Rights (Article 6) [11, 53]. In addition, the implications of the Helsinki Declaration of the World Medical Association [77] are also relevant, which in principle only permits medical research on people who have voluntarily consented to it and, which, in addition makes specifications for research on people who are incapable of giving consent. It is true that this declaration has no direct legal effect. Nevertheless, the Declaration has legal significance through references to and consideration of the principles it establishes for biomedical research in national and EU regulations [37]. Furthermore, informed consent is legally protected in the legislation of individual countries.

Regarding the legal requirements of informed consent, particular attention must be paid to possible differences between interventions for research purposes in the context of clinical studies, therapeutic application, and neuro-enhancement imaginable in the context of optogenetics. Relevant issues here include legal questions related to neural self-determination. In the area of neurology, self-determination is characterized on the one hand by the question of a person’s free (and informed) decision for (positive dimension) and on the other hand against (negative dimension) a possibly personality-changing intervention [79] on the central nervous system [35]. For example, in the context of deep brain stimulation [28, 49, 55], this is already a subject of jurisprudential discourse [36, 49]. A further challenge in the application of optogenetic methods is that, due to the severity or nature of their illness, potential subjects or patients may often have limited or no capacity to consent [72].

In the case of optogenetics, for example in comparison with deep brain stimulation methods, it should be noted in particular that the necessary genetic interventions are basically not reversible [72]. The irreversibility can also lead to the fact that an adaptation, and thus, a deviation from the usually internationally standardized phases of the clinical trial is legally required [72].

In any case, the question of the extent to which such an intervention can be consented to at all must also be considered in the light of the aspects of medical ethics already described in the ethical section of this article. Regarding optogenetic interventions on humans, it must therefore also be clarified to what extent the existing set of standards is adequate in terms of opportunities and risks, or whether changes to the law are necessary.

### Patent law

Furthermore, questions of patent law arise both in relation to cerebral organoids [19, 75, 80] and in relation to the technical processes of optogenetics. The answer to these questions requires an integrated assessment of the technical development from basic research to the application in humans. This is therefore also necessary regarding the successful translation of optogenetics from basic research to application. The reason for this is that the translation of a technology typically requires the patentability of the underlying invention in all phases of product development because this is the only way the inventor can be guaranteed exclusive commercial exploitation. Therefore, issues of patentability must be considered already in basic research—in which optogenetics is currently mostly conducted regarding an envisaged application in humans.

As part of a patent law investigation, it must be clarified, for example, to what extent and under what conditions inventions related to cerebral organoids can be patented [19, 80]. This raises questions such as the patentability of the cerebral organoids themselves [75] as well as the technical devices used. In this context, it might be necessary to differentiate between the current state of the development and more complex organoids that may be generated in the future [75].

Organoids generated from human embryonic stem cells (heSCs) are generally not patentable. For both the EU harmonized patent law and the patent regime of the European Patent Convention (EPC), patent protection for inventions based on heSCs is excluded because this use of heSCs has been considered contrary to the *ordre puplic* enshrined in patent law [7, 8, 17, 18]. In contrast, if the requirements for a patent are met, inventions for the generation of neuronal organoids derived from human induced pluripotent stem cells (hiPSCs) are in principle patentable [75], because neither explicit patent exclusions are relevant nor because even the commercial use of hiPSCs is not generally regarded as a violation of the *ordre public* principle [75].

However, regarding the therapeutic application of neuronal optogenetics, it must also be clarified whether at least certain uses of optogenetics such as targeted neuronal control and, based on this, a mental controllability, have an influence on possible patentability. First, it must be kept in mind that therapeutic methods as such cannot be patented (see Article 53(c) EPC, in Germany § 2a(1) No. 2 PatG); however, technical devices that are necessary for the therapy can be patented, provided that the patent requirements are met. Regarding the devices and objects that are patentable in principle, however, it must then again be taken into account that patent law can exclude the patentability of an invention by means of the *ordre public* exception (see Article 53(a) EPC; Article 6 of Directive 98/44/EC), considering ethical aspects [48, 75]. The extent to which the patentability of inventions of neuronal optogenetics can be restricted based on this patent exclusion will have to be clarified in the medico-ethical and legal discourse of the coming years.

## Outlook

It can be stated that from both an ethical and a legal perspective, that there is a need to clarify open questions of neuronal optogenetics. To enable the use of the medical potential of neuronal optogenetics, for which translation into clinical application is a prerequisite, it is necessary to bring the open ethical and legal questions into a broad and open discourse with all involved stakeholders.

## Data Availability

Not applicable.

## Declarations

### Open access

The declaration regarding a possible grant for open access publication can only be made after peer review.

## References

[1] Adamczyk AK, Zawadzki P (2020). The memory-modifying potential of optogenetics and the need for neuroethics. NanoEthics.

[2] Barnhart AJ, Dierickx K (2023) A tale of two chimeras: applying the six principles to human brain organoid xenotransplantation. Camb Q Healthc Ethics 1–17. 10.1017/S0963180123000051 10.1017/S0963180123000051 36847198

[3] Bernet Kempers E (2020). Animal dignity and the law: potential, problems and possible implications. Liverpool Law Rev.

[4] Birnbacher D (2006). Bioethik zwischen Natur und Interesse.

[5] Calliess C (2022) AEUV Art. 13 [Tierschutz; Querschnittsklausel]. In: Calliess C, Ruffert M (eds) EUV / AEUV - Kommentar, 6th ed. C.H. Beck, München, 535–538

[6] Chen HI, Wolf JA, Blue R, Song MM, Moreno JD, Ming G-L, Song H (2019). Transplantation of human brain organoids: revisiting the science and ethics of brain chimeras. Cell Stem Cell.

[7] Court of Justice of the European Union (CJEU) Oliver Brüstle v Greenpeace eV, JUDGMENT OF 18. 10. 2011, CASE C-34/10, ECLI:EU:C:2011:669

[8] Court of Justice of the European Union (CJEU) International Stem Cell Corporation v Comptroller General, JUDGMENT OF 18. 12. 2014, CASE C-364/13, ECLI:EU:C:2014:2451

[9] Damasio A (2012). Self comes to mind: constructing the conscious brain.

[10] Damasio AR (2005). Descartes’ error: emotion, reason, and the human brain.

[11] Dankar FK, Gergely M, Dankar SK (2019). Informed consent in biomedical research. Comput Struct Biotechnol J.

[12] Deubner J, Coulon P, Diester I (2019). Optogenetic approaches to study the mammalian brain. Curr Opin Struct Biol.

[13] Deuring S, Dederer H-G, Hamburger D (2022). The legal requirements for—and limits to—the donor’s and the patient’s consent. Brain organoids in research and therapy.

[14] Di Lullo E, Kriegstein AR (2017). The use of brain organoids to investigate neural development and disease. Nat Rev Neurosci.

[15] Forschungsgemeinschaft D (2017). Stellungnahme Tiefe Hirnstimulation.

[16] Edelman G, Tononi G (2000). A universe of consciousness: how matter becomes imagination.

[17] European Patent Office (EPO) T 1374/04 (Stem cells/WARF) 07–04–2006, ECLI:EP:BA:2006:T137404.20060407

[18] European Patent Office (EPO) G 0002/06 (Use of embryos/WARF) 25–11–2008, ECLI:EP:BA:2008:G000206.20081125

[19] Faltus T (2021). Organoide, Assembloide, Gastruloide, SHEEFs & Organ-on-Chip: Rechtliche und medizinethische Aspekte künstlich erzeugter Organ- und Embryomodelle. MedR.

[20] Garita-Hernandez M, Guibbal L, Toualbi L, Routet F, Chaffiol A, Winckler C, Harinquet M, Robert C, Fouquet S, Bellow S, Sahel J-A, Goureau O, Duebel J, Dalkara D (2018). Optogenetic light sensors in human retinal organoids. Front Neurosci.

[21] Gilbert F, Harris A, Kapsa R (2014) Controlling brain cells with light: ethical considerations for optogenetic clinical trials. Aust Inst Innov Mater - Pap 3–11. 10.1080/21507740.2014.911213

[22] Hee Lee J, Lee S, Kim D, Jae Lee K (2022). Implantable micro-light-emitting diode (µLED)-based optogenetic interfaces toward human applications. Adv Drug Deliv Rev.

[23] Hoppe N, Lorenz M, Teller J, Dederer H-G, Hamburger D (2022). Transplantation of human brain organoids into animals: the legal issues. Brain organoids in research and therapy.

[24] Ide K, Matsuoka N, Fujita M (2021). Ethical aspects of brain organoid research in news reports: an exploratory descriptive analysis. Medicina (Kaunas).

[25] Ienca M, Andorno R (2017). Towards new human rights in the age of neuroscience and neurotechnology. Life Sci Soc Policy.

[26] Jarass HD (2021). Charta der Grundrechte der Europäischen Union: unter Einbeziehung der sonstigen Grundrechtsregelungen des Primärrechts und der EMRK : Kommentar.

[27] Jaworska A (2017) Ethical dilemmas in neurodegenerative disease: respecting patients at the twilight of agency. In: Illes J (ed) Neuroethics: anticipating the future. Oxford University Press, p 0

[28] Katzenmeier C, Schmitz-Luhn B (2010). Rechtliche Aspekte der Tiefen-Hirnstimulation (Deep-Brain-Stimulation). Jurisprudenz zwischen Medizin und Kultur: Festschrift zum 70.

[29] Kersten J (2020) Die Rechte der Natur und die Verfassungsfrage des Anthropozän. In: Soentgen J, Gassner UM, Von Hayek J, Manzei A (eds) Umwelt und Gesundheit. Nomos, pp 87–120

[30] Keshmiri Neghab H, Soheilifar MH, Grusch M, Ortega MM, Esmaeeli Djavid G, Saboury AA, Goliaei B (2022). The state of the art of biomedical applications of optogenetics. Lasers Surg Med.

[31] Koplin JJ, Savulescu J (2019). Moral limits of brain organoid research. J Law Med Ethics.

[32] Lavazza A (2021). Potential ethical problems with human cerebral organoids: consciousness and moral status of future brains in a dish. Brain Res.

[33] Lavazza A, Dederer H-G, Hamburger D (2022). Human cerebral organoids: evolving entities and their moral status. Brain organoids in research and therapy.

[34] Legnini I, Emmenegger L, Zappulo A, Rybak-Wolf A, Wurmus R, Martinez AO, Jara CC, Boltengagen A, Hessler T, Mastrobuoni G, Kempa S, Zinzen R, Woehler A, Rajewsky N (2023). Spatiotemporal, optogenetic control of gene expression in organoids. Nat Methods.

[35] Lindner JF (2010). “Neuro-Enhancement” als Grundrechtsproblem. MedR.

[36] Lindner JF (2016). Die neuronale Selbstbestimmung des Menschen: Grundlagen und Gefährdungen.

[37] Lipp V (2021) Heilversuch und medizinische Forschung. In: Laufs A, Katzenmeier C, Lipp V (eds) Arztrecht, 8th ed. C.H. Beck, München

[38] MacDuffie KE, Stein JL, Doherty D, Jayadev S, Girault JB, Emmons KA, Glass MR, Dempsey JC, Marrus N, Botteron KN, Dager SR, Estes AM, Piven J, Wilfond BS (2023). Donor perspectives on informed consent and use of biospecimens for brain organoid research. Stem Cell Rep.

[39] Mahalatchimy A, Lau PL, Li P, Flear ML (2021). Framing and legitimating EU legal regulation of human gene-editing technologies: key facets and functions of an imaginary. J Law Biosci.

[40] Mayr S, Thiermann A, Schrack M, Kiesselbach C (2021). Das neue Medizinprodukterecht: Praxishandbuch zur MP-VO.

[41] Merkel R (2009). Neuartige Eingriffe ins Gehirn – Verbesserung der mentalen condicio humana und strafrechtliche Grenzen. ZStW.

[42] Miura Y, Li M-Y, Birey F, Ikeda K, Revah O, Thete MV, Park J-Y, Puno A, Lee SH, Porteus MH, Pașca SP (2020). Generation of human striatal organoids and cortico-striatal assembloids from human pluripotent stem cells. Nat Biotechnol.

[43] Muir J, Lopez J, Bagot RC (2019). Wiring the depressed brain: optogenetic and chemogenetic circuit interrogation in animal models of depression. Neuropsychopharmacol.

[44] Nagel G, Brauner M, Liewald JF, Adeishvili N, Bamberg E, Gottschalk A (2005). Light activation of channelrhodopsin-2 in excitable cells of Caenorhabditis elegans triggers rapid behavioral responses. Curr Biol.

[45] Najafabadi SH, Sadeghi M, Zibaii MI, Soheili Z-S, Samiee S, Ghasemi P, Hosseini M, Gholami Pourbadie H, Ahmadieh H, Taghizadeh S, Ranaei Pirmardan E (2021). Optogenetic control of neural differentiation in Opto-mGluR6 engineered retinal pigment epithelial cell line and mesenchymal stem cells. J Cell Biochem.

[46] Nixdorff K, Borisova T, Komisarenko S, Dando M (2018). Dual-use nano-neurotechnology: an assessment of the implications of trends in science and technology. Politics Life Sci.

[47] Ordaz JD, Wu W, Xu X-M (2017). Optogenetics and its application in neural degeneration and regeneration. Neural Regen Res.

[48] Osterrieth C (2021). Patentrecht.

[49] Prütting J (2014). Rechtliche Aspekte der Tiefen Hirnstimulation: Heilbehandlung, Forschung.

[50] Roth G (2005). Gehirn, Gründe und Ursachen. Dtsch Z Philos.

[51] Ryder RD (1971) Experiments on animals. In: Godlovitch S, Godlovitch R, Harris J (eds) Animals, man and morals: an enquiry into the maltreatment of non-humans. Gollancz, London

[52] Sahel J-A, Boulanger-Scemama E, Pagot C, Arleo A, Galluppi F, Martel JN, Esposti SD, Delaux A, de Saint Aubert J-B, de Montleau C, Gutman E, Audo I, Duebel J, Picaud S, Dalkara D, Blouin L, Taiel M, Roska B (2021). Partial recovery of visual function in a blind patient after optogenetic therapy. Nat Med.

[53] Salako SE (2011). Informed consent under the European Convention on Biomedicine and the UNESCO Declaration on Bioethics. Med Law.

[54] Schicktanz S, Bartfeld S, Schickl H, Alev C, Koo B-K, Pichl A, Osterheider A, Marx-Stölting L (2020). Sind menschliche zerebrale Organoide moralisch schützenswert? Ein kommentierter Überblick über die aktuelle internationale Ethikdiskussion. Organoide: Ihre Bedeutung für Forschung.

[55] Schmitz-Luhn B, Katzenmeier C, Woopen C (2012). Law and ethics of deep brain stimulation. Int J Law Psychiatry.

[56] Sekiguchi H, Matsuhira H, Kanda R, Tada S, Kitade T, Tsutsumi M, Nishikawa A, Loesing A, Fukunaga I, Setogawa S, Ohkawa N (2022). Adhesionable flexible GaN-based microLED array film to brain surface for in vivo optogenetic stimulation. Appl Phys Express.

[57] Sharma A, Zuk P, Scott C (2021). Scientific and ethical uncertainties in brain organoid research. Am J Bioeth.

[58] Shiri Z, Simorgh S, Naderi S, Baharvand H (2019). Optogenetics in the era of cerebral organoids. Trends Biotechnol.

[59] Soltan A, Barrett JM, Maaskant P, Armstrong N, Al-Atabany W, Chaudet L, Neil M, Sernagor E, Degenaar P (2018). A head mounted device stimulator for optogenetic retinal prosthesis. J Neural Eng.

[60] Song G, Zhao M, Chen H, Zhou X, Lenahan C, Ou Y, He Y (2021). The application of brain organoid technology in stroke research: challenges and prospects. Front Cell Neurosci.

[61] Stucki S (2016). Grundrechte für Tiere: Eine Kritik des geltenden Tierschutzrechts und rechtstheoretische Grundlegung von Tierrechten im Rahmen einer Neupositionierung des Tieres als Rechtssubjekt.

[62] Taupitz J (2020). Organoide. MedR.

[63] Taupitz J, Bartfeld S, Schickl H, Alev C, Koo B-K, Pichl A, Osterheider A, Marx-Stölting L (2020). Organoide: Die deutsche Rechtslage. Organoide.

[64] Taupitz J (2021). Der rechtliche Status von Hirnorganoiden. MedR.

[65] Taupitz J, Fehse B, Hucho F, Bartfeld S, Clemens S, Erb T, Fangerau H, Hampel J, Korte M, Marx-Stölting L, Mundlos S, Osterheider A, Pichl A, Reich J, Schickl H, Schicktanz S, Taupitz J, Walter J, Winkler E, Zenke M (2021). Humane Hirnorganoide: Die deutsche Rechtslage. Fünfter Gentechnologiebericht.

[66] Taupitz J, Dederer H-G, Hamburger D (2022). What Is, or Should Be, the Legal Status of Brain Organoids?. Brain organoids in research and therapy: fundamental ethical and legal aspects.

[67] Trujillo CA, Gao R, Negraes PD, Gu J, Buchanan J, Preissl S, Wang A, Wu W, Haddad GG, Chaim IA, Domissy A, Vandenberghe M, Devor A, Yeo GW, Voytek B, Muotri AR (2019). Complex oscillatory waves emerging from cortical organoids model early human brain network development. Cell Stem Cell.

[68] Vajtay TJ, Bandi A, Upadhyay A, Swerdel MR, Hart RP, Lee CR, Margolis DJ (2019). Optogenetic and transcriptomic interrogation of enhanced muscle function in the paralyzed mouse whisker pad. J Neurophysiol.

[69] Voeneky S, Dederer H-G, Hamburger D (2022). Global Harmonization of Legal Standards for Brain Organoid Research and Therapy?. Brain organoids in research and therapy: fundamental ethical and legal aspects.

[70] Walter H, Müller S, Hegemann P, Sigrist S (2013). Optogenetics as a new therapeutic tool in medicine? A view from the principles of biomedical ethics. Optogenetics.

[71] Wentz CT, Oettl L-L, Kelsch W (2013). Optogenetics in psychiatric animal models. Cell Tissue Res.

[72] White M, Mackay M, Whittaker RG (2020). Taking optogenetics into the human brain: opportunities and challenges in clinical trial design. OAJCT.

[73] White M, Whittaker RG (2022). Post-trial considerations for an early phase optogenetic trial in the human brain. OAJCT.

[74] Wiese L (2022). Hirnorganoide als (potentielle) „Novel Beings“ — Ein Plädoyer für eine frühzeitige und interdisziplinäre Debatte mit Weitblick. GesR.

[75] Wolff H (2022) Patentierbarkeit von aus iPSZ hergestellten Gehirnorganoiden. GRUR 1473–1481

[76] Wolff H, Kunz A (2021). Hirnorganoide – Ethische Herausforderungen und ihre Berücksichtigung und Umsetzung im Recht. MedR.

[77] World Medical Association (2013). Declaration of Helsinki: ethical principles for medical research involving human subjects. JAMA.

[78] Yuste R, Genser J, Herrmann S (2021) It’s time for neuro-rights: new human rights for the age of neurotechnology. Hor J Int Relat Sustain Dev 154–165

[79] Zawadzki P, Adamczyk AK (2021). Personality and authenticity in light of the memory-modifying potential of optogenetics. AJOB Neurosci.

[80] Zhu L, Fan Y, Huang X, Chen T, Xu X, Xu F, Gong Y, Chen P (2022). Patent bibliometric analysis for global trend of organoid technologies in the past decade. iScience.

[81] Zillmann H, Kaufmann M, Faltus T (2019). Genomeditierung. Ethische Bewertung der verschiedenen Anwendungsbereiche. Ethik, Recht und Kommunikation des Genome Editings.

